# Early postoperative patient-reported outcomes in thoracoscopic segmentectomy: a comparative study of non-intubated anesthesia versus intubated general anesthesia

**DOI:** 10.3389/fonc.2025.1602812

**Published:** 2025-07-07

**Authors:** He Guan, Jinchao Bi, Wantong Zheng, Yahao Zhang, Zhijun Han, Li Wei, Jiwei Li

**Affiliations:** ^1^ Department of Thoracic Surgery, Zhengzhou University People’s Hospital, Henan Provincial People’s Clinical Medical School of Zhengzhou University, Zhengzhou, Henan, China; ^2^ Department of Thoracic Surgery, Henan Provincial People’s Hospital, Zhengzhou, Henan, China

**Keywords:** patient-reported outcomes, thoracoscopic surgery, segmentectomy, non-intubated anesthesia, intubated general anesthesia

## Abstract

**Introduction:**

Segmentectomy under non-intubated anesthesia (NIA) has demonstrated comparable conventional clinical outcomes to segmentectomy performed under intubated general anesthesia (IGA). However, differences in early patient-reported outcomes (PROs) between the two anesthetic approaches remain unclear. This study aimed to evaluate symptom burden and functional status from the patient’s perspective under different anesthesia modalities.

**Methods:**

Patients who underwent segmentectomy via either IGA or NIA were included. Perioperative symptom severity and functional status were assessed using the PSA-lung scale. PROs data were collected at various perioperative time points, and comparisons between groups were analyzed using a linear mixed-effects model.

**Results:**

Among the 380 enrolled patients, 160 underwent segmentectomy under NIA, and 220 under IGA. After propensity score matching (PSM), baseline characteristics were comparable between groups. On postoperative day 7, patients in the NIA group reported significantly milder symptoms of pain (P<0.001), cough (P<0.001), dyspnea (P=0.011), and drowsiness (P<0.001) compared to those in the IGA group. Additionally, the NIA group experienced less functional interference in walking (P<0.001) and general function (P<0.001). Within one month postoperatively, patients in the IGA group reported more severe cough (P<0.001) and anxiety (P<0.001) than those in the NIA group. There were no significant differences in short-term clinical outcomes between the two groups, although the NIA group had a longer operative time (P<0.001) but a shorter postoperative hospital stay (P<0.001).

**Discussion:**

PROs are essential indicators of postoperative recovery after segmentectomy. Compared to intubated anesthesia, non-intubated anesthesia is associated with fewer severe early symptoms, lower functional burden, and shorter hospitalization following segmentectomy.

## 1 Introduction

Lung cancer remains one of the most prevalent malignancies worldwide, imposing significant health and economic burdens (1). In recent years, advancements in imaging technologies and heightened public health awareness have led to increased early detection rates of lung cancer (2). Studies have demonstrated that, for patients with non-small cell lung cancer characterized by tumors ≤2 cm and a consolidation-to-tumor ratio between 0.5 and 1, segmentectomy offers comparable outcomes to lobectomy in terms of complications, mortality, and five-year overall survival rates (3). Moreover, segmentectomy preserves more lung parenchyma, making it a preferable option for many patients (4). Consequently, segmentectomy has become the treatment of choice for an increasing number of patients with early-stage NSCLC.

Traditionally, video-assisted thoracoscopic segmentectomy has been performed under IGA in major medical centers. This approach ensures satisfactory intraoperative ventilation and lung isolation, facilitating safe and efficient thoracic surgical procedures (5). However, endotracheal intubation can lead to adverse effects such as tracheal injury, laryngeal edema, and hoarseness (6–8). With advancements in anesthetic techniques and surgical proficiency, NIA has garnered attention. The spontaneous breathing NIA technique is increasingly applied in various thoracoscopic procedures (9–11). Existing literature suggests that NIA achieves clinical outcomes comparable to those of IGA (11–14). However, from the patient’s perspective, traditional clinical endpoints may not fully capture the postoperative experience and recovery quality associated with the two anesthetic approaches.

PROs are direct reports from patients regarding their health status, functional well-being, treatment experiences, and the impact on daily life. Numerous studies have shown that symptom monitoring based on PROs can alleviate symptom burden, enhance treatment responsiveness, improve prognosis, and facilitate better patient-provider communication (15–17). As a result, the FDA recommends PROs as novel clinical outcome indicators (18). PROs measurement tools are specialized instruments designed to assess PROs accurately, reflecting patients’ actual conditions, including symptom severity and functional status. They help identify perceived differences between seemingly similar surgical procedures, offering new insights into optimal surgical practices and providing scientific evidence for intervention optimization.

Currently, the symptom and functional burdens experienced by patients undergoing thoracoscopic segmentectomy under different anesthetic modalities remain unclear, particularly in the early postoperative period. This study aims to investigate early postoperative PROs and recovery quality following segmentectomy under different anesthesia methods through frequent, repeated assessments. The findings will provide a basis for anesthesiologists and surgeons to optimize perioperative management and inform clinical decision-making.

## 2 Materials and methods

### 2.1 Study design and population

We conducted a retrospective analysis using the database from a prospective longitudinal observational cohort study performed at a single center (Zhengzhou University People’s Hospital) between February 2022 and December 2024. This study was approved by the Ethics Committee of Zhengzhou University People’s Hospital, and all participants provided written informed consent. All methods were carried out in accordance with relevant guidelines and regulations.

Patients who underwent video-assisted thoracoscopic segmentectomy for malignant pulmonary lesions by the same surgical team were included. Inclusion criteria were: (a) age ≥18 years; (b) ability to tolerate and consent to segmentectomy under either NIA or IGA; (c) American Society of Anesthesiologists classification ≤ II; (d) diagnosis of lung cancer (The staging was based on the ninth version of lung cancer); (e) ability to understand study requirements and willingness to participate. Exclusion criteria included: (a) contraindications to the anesthetic or surgical techniques; (b) history of other malignancies; (c) previous thoracic surgery; (d)require conversion to open chest surgery; (e)transfer to intensive care unit; (f) inability to comprehend the study content.

**Figure 1:**
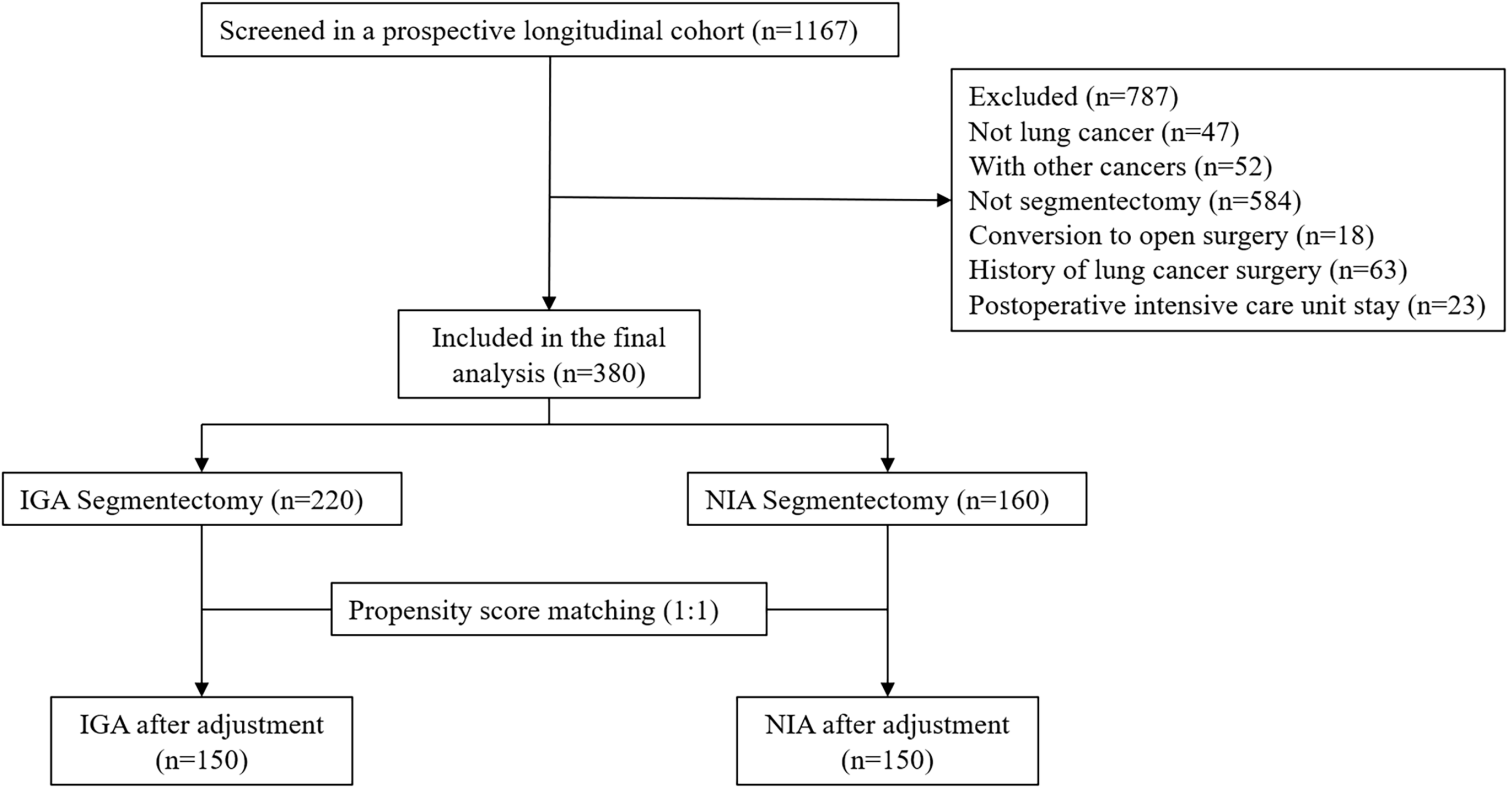
Patients selection. (IGA, intubated general anesthesia; NIA, non-intubated anesthesia; PSM, propensity score matching).

To ensure patient safety, conversion from NIA to conventional IGA was performed if any of the following intraoperative risk situations occurred:​ (a) Extensive and dense pleural adhesions within the thoracic cavity leading to poor surgical field exposure, significant surgical trauma, or uncontrolled bleeding; (b) Unsatisfactory lung collapse affecting the surgical procedure; (c) Inability to maintain adequate intraoperative oxygenation despite assisted ventilation, accompanied by carbon dioxide retention; (d) Tachypnea, significant increase in airway secretions, or sudden rise in airway pressure; (e) Severe hypotension, malignant arrhythmias, or other life-threatening conditions (**Figure 1**).

### 2.2 Surgical procedure and postoperative care

Each patient was decided by the anesthesiologist before the operation based on core indicators such as the patient’s age, type of surgery, and airway conditions to ensure that each patient included in the study could tolerate NIA or IGA. Meanwhile, before the operation, the surgeon will review the patient’s relevant examinations and the results of three-dimensional lung reconstruction to assess whether the operation can proceed smoothly under different anesthesia methods. If unexpected situations such as thoracic adhesions occur during the operation, they should be promptly switched to IGA anesthesia and excluded in this study.​NIA Group: Oxygenation was maintained via face mask (SpO_2_ >90%). Sedation and analgesia were achieved with low-dose fentanyl combined with propofol to maintain a bispectral index between 40-60. Neuraxial blockade involved local infiltration at the incision site combined with intercostal nerve blocks (incision site infiltration plus 0.5% bupivacaine for intercostal nerve blocks at levels L3-8, 1.5 ml per point). Under thoracoscopic guidance, vagus nerve blockade (3 ml of 0.5% bupivacaine) was performed to suppress cough reflex. ​(Nerve block causes less trauma and has a lower risk of infection. In contrast, the anesthesia level of epidural anesthesia is elevated, affecting respiratory and circulatory functions. Bilateral analgesia can also paralyze the intercostal muscles, which is not conducive to postoperative breathing, expectoration and rapid recovery.) IGA Group: Routine induction and maintenance of muscle relaxation were achieved with rocuronium, and sedation and analgesia were maintained with fentanyl and propofol. Intercostal nerve blocks were performed as in the NIA group. Patients received 100% oxygen throughout the procedure. Airway management involved the use of a double-lumen endotracheal tube or a single-lumen tube combined with a bronchial blocker. Ventilation parameters were set with tidal volumes of 6–8 ml/kg during two-lung ventilation and 4–6 ml/kg during one-lung ventilation, with a positive end-expiratory pressure of 5 cm H_2_O. Respiratory rate was adjusted to maintain end-tidal carbon dioxide between 35–45 mmHg.

Segmentectomy was performed using a two-port video-assisted thoracoscopic surgery technique. The main operating port was located at the 4th or 5th intercostal space, approximately 3 cm in length, while the thoracoscopic observation port was at the 7th intercostal space, about 1.5 cm in length. Based on tumor location, procedures were classified as segmentectomy or combined segmentectomy. Combined segmentectomy was indicated for lesions located at the boundaries of two or more segments, necessitating resection of adjacent segments to ensure adequate surgical margins. Systematic lymph node dissection was defined as the removal of three or more N2 lymph nodes (including station 7) and three or more N1 lymph nodes. Lymph node stations were categorized according to the International Association for the Study of Lung Cancer lymph node map (19). Procedures not meeting these criteria were classified as selective lymph node sampling. Intraoperative use of incision protectors and staplers was routine. At the end of the surgery, a chest tube was placed through the thoracoscopic observation port.

All patients received standardized postoperative care. Stool softeners were given to each patient when needed, and propacetamol for analgesia was administered twice a day after the operation. The urinary catheter was removed 24 or 48 hours after the operation and the patient was encouraged to get out of bed and move around. In addition, each person is required to carry a pain relief pump on a regular basis (available on demand). Each patient was given ipratropium bromide combined with budesonide nebulization treatment twice a day. Mechanical assisted expectoration was given twice a day after the operation. Chest tubes were removed when chest radiographs confirmed adequate lung expansion, absence of air leaks, and drainage volume less than 200 ml over 24 hours.

### 2.3 Outcomes and measures

The primary outcome of this study was to assess the severity of early postoperative symptoms and functional status as reported by patients. Evaluations were conducted using the Perioperative Symptom Assessment for Lung Surgery (PSA-Lung) scale, a tool recognized for its sensitivity, specificity, and reliability in assessing perioperative symptoms in lung surgery patients (20). The PSA-Lung scale comprises seven symptom items and two functional items; its validity and reliability were presented at the 2021 International Society for Quality of Life Research conference (21). Each item on the PSA-Lung is scored on a scale from 0 to 10, with higher scores indicating worse outcomes. PROs data were collected via electronic PSA-Lung questionnaires. Each patient completed the PSA-Lung electronic questionnaire once preoperatively, daily for the first seven postoperative days, and weekly for four weeks thereafter. PROs data were primarily collected through paper or electronic questionnaires.

Secondary outcomes included short-term clinical results such as operative time, number of lymph node stations dissected, drainage volume, length of postoperative hospital stay, and postoperative complications (Prolonged air leak, pneumonia, pulmonary embolism, chylothorax, arrhythmia). The Clavien-Dindo classification (22) was used to assess postoperative complications occurring within 4 weeks. Additionally, demographic and baseline characteristic data of the patients were collected.

### 2.4 Statistical analysis

Participants who completed all PSA-Lung questionnaires were included in the analysis, focusing on PROs data collected preoperatively, daily for the first seven postoperative days, and weekly up to four weeks post-discharge. Cases with missing PSA-Lung questionnaire data were excluded.

**Figure 2:**
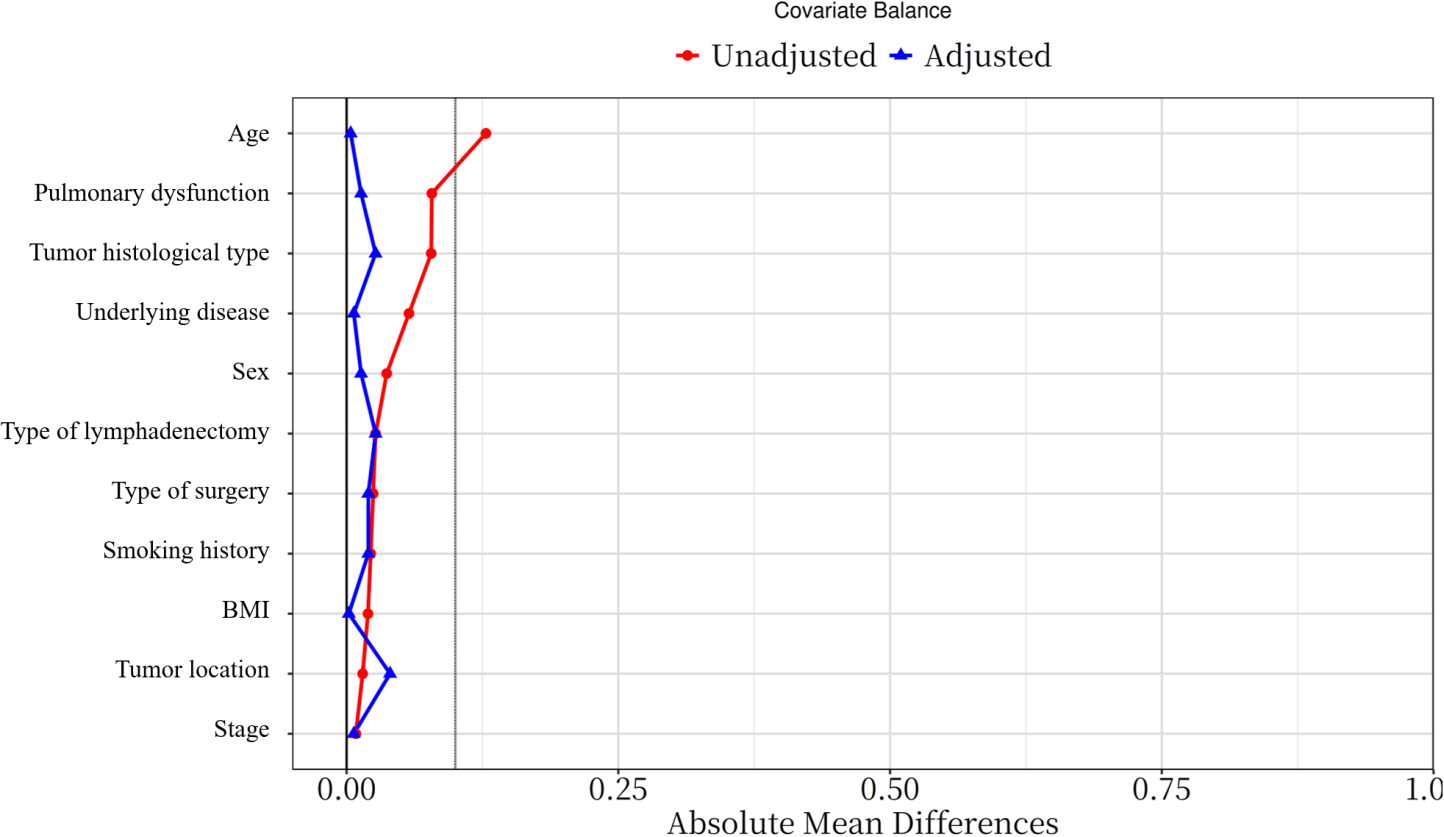
Standardized mean difference of variables before and after PSM (PSM: propensity score matching).

To mitigate potential biases, PSM was performed using the Match package in R. Patients undergoing NIA were matched to those receiving IGA based on variables including gender, age, BMI, comorbidities, smoking status, tumor location, surgical type, pulmonary function, histological type, pathological stage, and lymphadenectomy type. A caliper width of 0.1 times the standard deviation of the propensity score was applied to enhance baseline covariate balance. Matching was conducted at a 1:1 ratio using nearest neighbor matching without replacement. The effectiveness of PSM was assessed by standardized mean differences (SMD), with SMD ≤ 0.1 indicating adequate balance (**Figure 2**).

Descriptive statistics were used to summarize demographic and clinical characteristics, stratified by anesthesia type. Continuous variables were analyzed using t-tests for normally distributed data and Wilcoxon rank-sum tests for non-normally distributed data. Categorical variables were compared using chi-square tests.​ A linear mixed-effects model was employed to analyze changes in PROs scores over time between the NIA and IGA groups. Patient group, time (days or weeks following surgery), and the interaction between patient group and time were classified as fixed effects. Subject and time were classified as random effects. Parameter estimation utilized maximum likelihood estimation, adjusting for potential confounders. Statistical significance was set at P < 0.05.​ Statistical analyses were conducted using IBM SPSS Statistics version 26.0 and R software version 4.3.3. Graphs were generated using GraphPad Prism version 9.0.

## 3 Result

### 3.1 Patient characteristics

A total of 380 patients were included in the final analysis, with a mean age of 55.97 years; 246 patients (64.74%) were female. Among them, 160 and 220 patients underwent video-assisted thoracoscopic segmentectomy under NIA and IGA, respectively. After 1:1 PSM, 300 patients were included. Post-matching, there were no significant differences between the groups concerning age, BMI, smoking history, pulmonary function (pulmonary ventilation dysfunction, pulmonary diffusion dysfunction, or a combination of both in the preoperative pulmonary function examination), tumor location, type of surgery, sex, stage, underlying disease (non-surgical taboo of chronic diseases, including: Hypertension, coronary heart disease, diabetes, old cerebral infarction, peptic ulcer, etc.), lymphadenectomy type, pathological type (**Table 1**).

**Table 1:** Patient demographics and clinical characteristics.

Variables	Before matching			After PSM		
	NIA (n=160)	IGA (n=220)	P	NIA (n=150)	IGA (n=150)	P
Age	55.02 ± 10.56	56.61 ± 12.40	0.18	55.11 ± 10.88	55.06 ± 11.64	0.97
BMI	23.82 ± 3.08	23.88 ± 3.22	0.19	23.77 ± 3.08	23.76 ± 3.18	0.99
Smoking history			0.61			0.68
–	125 (78.12)	167 (75.91)		116 (77.33)	113 (75.33)	
+	35 (21.88)	53 (24.09)		34 (22.67)	37 (24.67)	
Pulmonary dysfunction			0.12			0.81
–	110 (68.75)	134 (60.91)		100 (66.67)	102 (68.00)	
+	50 (31.25)	86 (39.09)		50 (33.33)	48 (32.00)	
Tumor location			0.75			0.43
UL	118 (73.75)	159 (72.27)		109 (72.67)	115 (76.67)	
NUL	42 (26.25)	61 (27.73)		41 (27.33)	35 (23.33)	
Type of surgery			0.76			0.71
Single	103 (64.38)	145 (65.91)		104 (69.33)	101 (67.33)	
Combined	57 (35.62)	75 (34.09)		46 (30.67)	49 (32.67)	
Sex			0.46			0.81
Male	53 (33.12)	81 (36.82)		51 (34.00)	53 (35.33)	
Female	107 (66.88)	139 (83.18)		99 (66.00)	97 (64.67)	
Underlying disease			0.24			0.90
–	111 (69.38)	140 (63.64)		103 (68.67)	104 (69.33)	
+	49 (30.63)	80 (36.36)		47 (31.33)	46 (30.67)	
Stage			0.86			1.00
I	158 (98.75)	215 (97.73)		147 (98.00)	146 (97.33)	
II	2 (1.25)	5 (2.27)		3 (2.00)	4 (2.67)	
Type of lymphadenectomy			0.49			0.62
SLND	107 (66.88)	153 (69.55)		102 (68.00)	106 (70.67)	
SND	53 (33.12)	67 (30.45)		48 (32.00)	44 (29.33)	
Tumor histological type			0.023			0.47
Non-adenocarcinoma	27 (16.88)	20 (9.09)		19 (12.67)	15 (10.00)	
Adenocarcinoma	133 (83.12)	200 (90.91)		131 (87.33)	135 (90.00)	

BMI, Body Mass Index; IGA, intubated general anesthesia; NIA, non-intubated anesthesia; UL, upper lobe; NUL, non upper lobe; SLND, selective lymph node dissection; SND, systematic nodal dissection; + = yes; - = no.

### 3.2 PROs date overview

**Figure 3:**
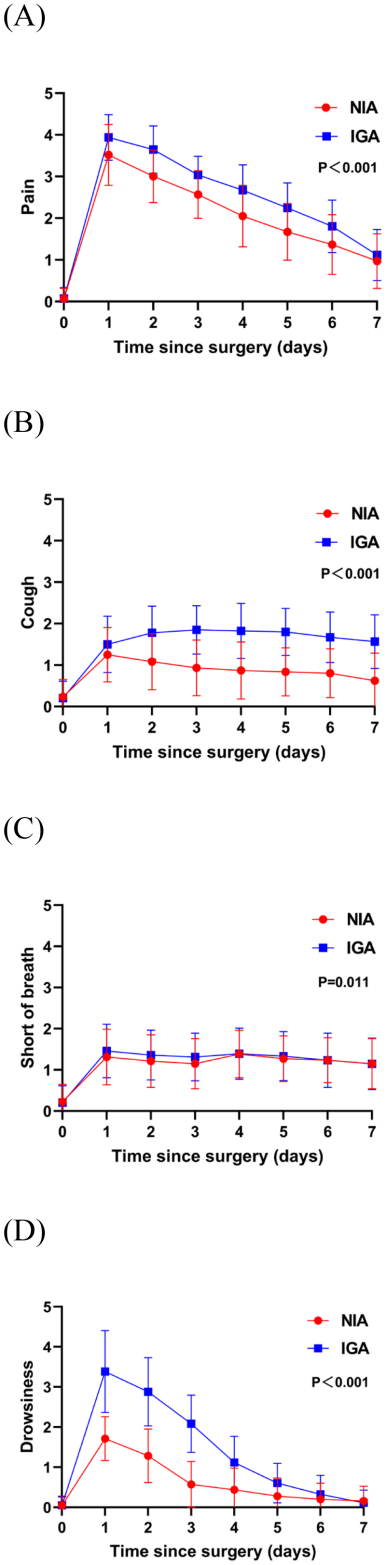
Severity of symptoms reported by patients during the 7-day postoperative period. **(A)** The score for the pain symptom; **(B)** the score for cough symptom; **(C)** the score for short of breath; **(D)** the score for drowsiness symptoms. (IGA, intubated general anesthesia; NIA, non-intubated anesthesia).

**Figure 4:**
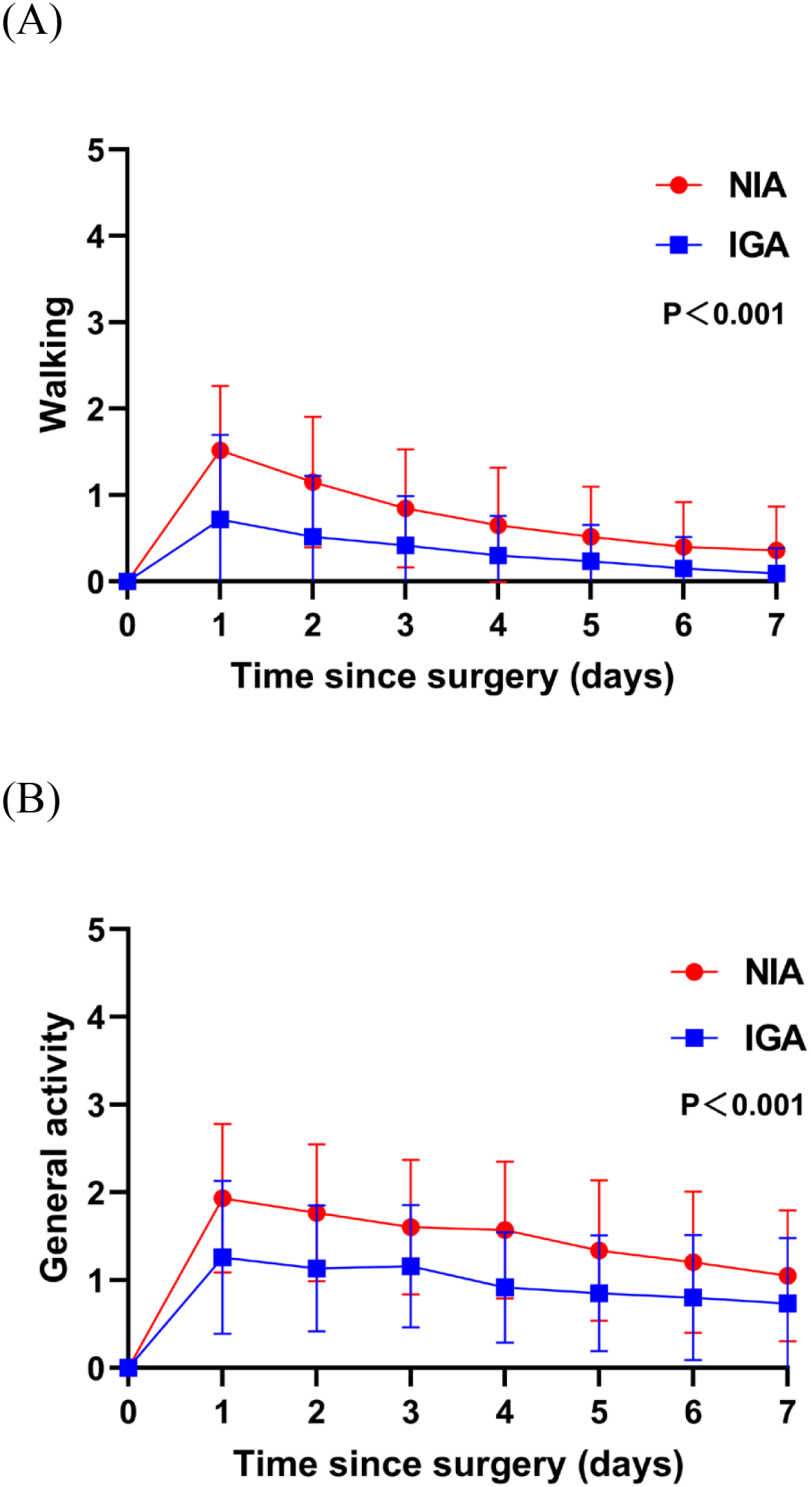
Functional status of patient-reported outcomes during the 7-day postoperative period. **(A)** The score for walking function; **(B)** the score for general activity function. (IGA, intubated general anesthesia; NIA, non-intubated anesthesia).

**Figure 5:**
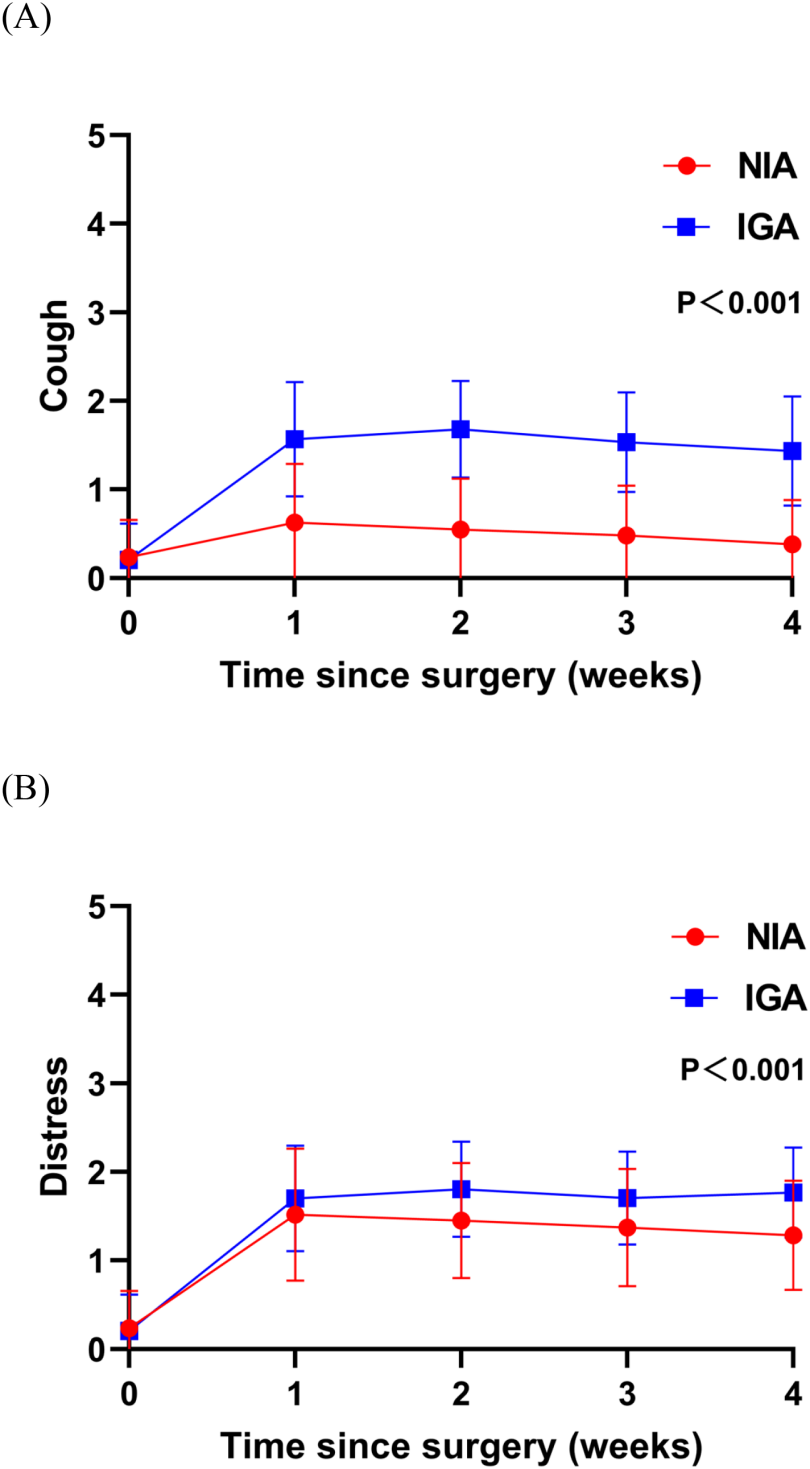
Severity of symptoms reported by patients during the 4-week postoperative period. **(A)** The score for the cough symptom; **(B)** the score for distress symptom. (IGA, intubated general anesthesia; NIA, non-intubated anesthesia).

At baseline, no significant differences were observed in symptoms or functional status between the groups. On postoperative day 7, patients in the NIA group reported significantly less severe symptoms (**Figure 3**), including pain, cough, shortness of breath, and drowsiness. Additionally, compared to the IGA group, NIA patients exhibited less functional impairment in walking and general activities (**Figure 4**). No significant differences were noted between the groups regarding distress, disturbed sleep, fatigue symptoms. Within four weeks post-discharge, symptom severity and functional status were comparable between the NIA and IGA groups, except for cough and distress, which remained less severe in the NIA group (**Figure 5**).

### 3.3 Traditional clinical outcomes

A comparison of surgery-related outcomes between the different anesthesia methods is presented in **Table 2**. No significant differences were found between the groups in terms of intraoperative blood loss (P=0.88 before matching; P=0.82 after PSM), number of lymph node stations dissected (P=0.33 before matching; P=0.31 after PSM), or postoperative complications (P=0.65 before matching; P=0.86 after PSM), with all postoperative complications classified as Clavien-Dindo grade ≤ II. After balancing baseline characteristics, the NIA group exhibited a trend toward reduced postoperative drainage volume (P=0.041 before matching; P=0.012 after PSM). However, compared to the IGA group, NIA patients had longer operative times (P<0.001 before matching; P<0.001 after PSM) and shorter hospital stays (P<0.001 before matching; P<0.001 after PSM).

**Table 2:** Traditional clinical outcomes.

Variables	Before matching			After PSM		
	NIA (n=160)	IGA (n=220)	P	NIA (n=150)	IGA (n=150)	P
Intraoperative hemorrhage	34.88 ± 26.44	34.48 ± 26.16	0.88	35.13 ± 26.51	34.67 ± 26.79	0.88
Operative time	167.67 ± 46.73	146.83 ± 44.44	**<0.001**	167.29 ± 46.53	146.64 ± 44.88	**<0.001**
Dissected of LN stations	5.04 ± 1.89	4.86 ± 1.64	0.33	5.00 ± 1.90	4.81 ± 1.56	0.35
Chest tube drainage	522.94 ± 321.38	587.20 ± 286.73	**0.041**	517.60 ± 319.62	606.50 ± 290.25	**0.012**
Postoperative length of stay	3.66 ± 1.56	5.52 ± 2.12	**<0.001**	3.67 ± 1.58	5.61 ± 2.19	**<0.001**
Postoperative complications			0.65			0.61
–	140 (87.50)	189 (85.91)		131 (87.33)	128 (85.33)	
+	20 (12.50)	31 (14.09)		19 (12.67)	22 (14.67)	
Prolonged air leak			0.75			0.40
–	149 (93.12)	203 (92.27)		140 (93.33)	136 (90.67)	
+	11 (6.88)	17 (7.73)		10 (6.67)	14 (9.33)	
Pneumonia			0.65			0.50
–	157 (98.12)	213 (96.82)		147 (98.00)	144 (96.00)	
+	3 (1.88)	7 (3.18)		3 (2.00)	6 (4.00)	
Chylothorax			1.00			1.00
–	156 (97.50)	214 (97.27)		146 (97.33)	147 (98.00)	
+	4 (2.50)	6 (2.73)		4 (2.67)	3 (2.00)	
Pulmonary embolism			0.51			0.48
–	160 (100.00)	218 (99.09)		150 (100.00)	148 (98.67)	
+	0 (0.00)	2 (0.91)		0 (0.00)	2 (1.33)	
Arrhythmia			1.00			1.00
–	158 (98.75)	217 (98.64)		148 (98.67)	149 (99.33)	
+	2 (1.25)	3 (1.36)		2 (1.33)	1 (0.67)	

IGA, intubated general anesthesia; NIA, non-intubated anesthesia; + = yes; - = no; LN, lymph node.

The bold values indicate that there is a statistically significant difference between the two groups.

## 4 Discussion

Traditional clinical metrics have often been employed to evaluate the advantages and disadvantages of different surgical approaches, frequently overlooking patient experiences and perceptions. Attentively considering patients’ viewpoints not only embodies fundamental medical ethics but also serves as a crucial means to enhance healthcare services and patient satisfaction. PROs have recently gained attention as valuable indicators for assessing postoperative recovery in lung cancer surgeries, offering deeper insights into patient-centered rehabilitation post-surgery (23–26). Concurrently, NIA is increasingly utilized worldwide, extending to complex procedures such as radical lung cancer surgeries and even lung transplants (10), its advantages over IGA from the perspective of PROs have yet to be definitively established. To our knowledge, this study is an earlier to evaluate PROs between NIA and IGA in patients undergoing segmentectomy. Our findings indicate comparable outcomes between the two groups, with NIA patients experiencing lower symptom burdens and better functional status postoperatively compared to those in the IGA group.

PROs following segmentectomy are influenced by multiple factors, including patient characteristics and surgical variables. In this study, preoperative characteristics were similar between groups. The early advantages of NIA in PROs may reflect reduced airway trauma and decreased use of anesthetic agents, leading to less physiological and functional disruption. Tracheal intubation and mechanical ventilation can directly irritate the pharyngeal and tracheal mucosa, resulting in postoperative throat pain and cough. Prolonged mechanical ventilation may cause alveolar overdistension and inflammatory responses (ventilator induced lung injury), exacerbating postoperative chest discomfort and pain. Additionally, IGA requires higher doses of sedatives and neuromuscular blockers, with delayed drug metabolism potentially prolonging postoperative sedation and indirectly intensifying pain perception. By preserving spontaneous breathing and incorporating local anesthesia, NIA significantly reduces postoperative pain perception. Pain in IGA patients may limit postoperative respiratory exercises, exacerbating symptoms of shortness of breath. The increased anesthetic requirements in IGA patients elevate the risk of postoperative complications such as restless sleep, fatigue, and drowsiness.​ Furthermore, reduced pain and dyspnea may, in turn, lessen impairment in walking and general activity functions.

Compared to patients undergoing IGA, those receiving NIA exhibit better postoperative mobility and general functional status. Early ambulation is fundamental to rapid recovery after thoracic surgery. Numerous studies have demonstrated the efficacy of early mobilization in reducing postoperative complications and accelerating functional recovery, including the clearance of airway secretions, decreasing the risks of atelectasis and pneumonia, and venous thrombosis (27–29). Early activity can expedite respiratory function recovery, stimulate deep breathing and coughing, enhance forced vital capacity, and reduce dependence on oxygen. Additionally, early mobilization offers psychological benefits, such as reduced perception of pain.

Notably, during follow-up after discharge, we observed no significant differences between the NIA and IGA groups in symptom severity and functional interference, except for cough and anxiety. This suggests that the impact of NIA on PROs tends to diminish over time. Persistent postoperative cough is a common complication following lung cancer surgery. Although less life-threatening than complications like progressive hemothorax, pneumothorax, and chylothorax, persistent cough can increase psychological burden, leading patients to question the efficacy of surgery and potentially causing anxiety and depression (30, 31). This may explain the more severe postoperative anxiety observed in IGA patients. Further characterization of persistent postoperative cough and early intervention in high-risk populations will be focal points of our subsequent research.

Several studies have demonstrated that NIA significantly shortens postoperative recovery room stays, time to ambulation, hospital length of stay, and overall hospitalization costs, indicating a positive impact on postoperative recovery for VATS patients (32, 33). In our study, the NIA group exhibited comparable intraoperative blood loss, number of lymph node stations dissected, postoperative drainage volume, and complication rates to the IGA group. Compared with the complete collapse of the affected lung in the IGA group, the affected lung in the NIA group had a certain degree of mobility and the surgical field was not good. To avoid lung traction injury, the respiratory circuit needs to be frequently disconnected to cooperate with thoracoscopic operations. However, tracheal intubation for one-lung ventilation can directly achieve lung collapse and reduce intraoperative pauses. Meanwhile, during tube-free anesthesia, the face mask is prone to displacement due to changes in body position (such as lateral position) or fluctuations in airway pressure. All the above reasons may increase the operation time. The NIA procedure minimizes the adverse effects of tracheal intubation and general anesthesia to the greatest extent, such as airway trauma related to intubation, lung injury caused by ventilation, residual neuromuscular block, and postoperative nausea and vomiting (34, 35). After the operation, patients under NIA have a significantly reduced risk of postoperative respiratory muscle paralysis due to a lower dosage of muscle relaxants during the operation, retention of diaphragm movement (36), better respiratory and expectoration functions, accelerated postoperative recovery, and shortened hospital stay. Furthermore, during one-lung ventilation with IGA, positive pressure ventilation may lead to excessive alveolar expansion and barotrauma, increasing the risk of exudation and air leakage in the surgical side of the lung (37, 38). The systemic inflammatory response caused by tracheal intubation may aggravate pleural exudation (27). NIA reduces the shear force of the lung parenchyma and the postoperative thoracic drainage volume by preserving spontaneous breathing or low tidal volume strategies, which is similar to the conclusions of previous studies (39). Although NIA significantly increased operative time, it resulted in better short-term recovery outcomes and shorter hospital stays, enhancing the overall patient experience.

Compared to previous studies comparing NIA and IGA, our research offers new insights. Firstly, NIA demonstrates good safety and reliability in moderately complex lung surgeries such as segmentectomy. Secondly, beyond traditional short-term surgical outcomes, this study employs PROs as indicators of short-term prognosis following segmentectomy, revealing lower symptom and functional burdens in NIA patients postoperatively. Thirdly, unlike earlier studies focusing primarily on pain, this research includes nine postoperative symptoms and functional items, providing a more comprehensive assessment of the postoperative experience, which is vital for delivering patient-centered care. Fourthly, we collected PROs at multiple postoperative time points, highlighting the severity and variability of early postoperative experiences following segmentectomy. Collecting PROs data offers clinicians more robust evidence for patient condition assessment, contributing to improved quality of life, enhanced recovery, strengthened doctor-patient communication, and reduced emergency department utilization (40). While some studies suggest that NIA offers long-term benefits over IGA (41, 42), this conclusion requires further investigation through large-scale randomized controlled trials to develop safer, more effective, and less invasive surgical strategies for patient optimization.

This study has certain limitations. Firstly, collecting PRO data weekly after discharge may not fully capture the dynamic changes in postoperative symptoms and functional status, particularly in the early postoperative period. Secondly, as a single-center study conducted in China, the generalizability of our findings is limited. We are seeking to conduct randomized controlled trials to validate our conclusions. Thirdly, only patients with ASA ≤ 2 were included in this study, which limited the universality of the study. Subsequently, we will gradually attempt to conduct research in a broader patient population. Fourthly, the retrospective nature of this study may introduce potential biases. For instance, surgeons may prefer IGA for patients undergoing complex segmentectomies due to perceived safety. Although baseline characteristics were balanced before analysis, large-scale randomized controlled trials are needed in the future to draw definitive conclusions. Fifthly, at present, the guidelines recommend reducing the oxygen concentration during the ventilation maintenance period to reduce the occurrence of complications such as postoperative oxygenation dysfunction. Due to the certain era limitations of the research design and certain conflicts with the current guidelines, this is one of the limitations of the article.

## 5 Conclusion

In conclusion, segmentectomy under NIA yields comparable short-term clinical outcomes to IGA, with faster recovery and lighter early postoperative symptom burdens, as well as better functional status. Future research is needed to elucidate the potential long-term effects of NIA on postoperative recovery and tumor recurrence in segmentectomy patients.

## Data Availability

The raw data supporting the conclusions of this article will be made available by the authors, without undue reservation.
